# Supplementary material for: A UMLS-based spell checker for natural language processing in vaccine safety
Source: BMC Med Inform Decis Mak. 2007 Feb 12;7:3. doi: 10.1186/1472-6947-7-3 (PMC1805499; doi:10.1186/1472-6947-7-3)
Supplement: Additional file 13 — RAP application directory. Sets up directory for RAP (RDF application for PHP) source code [file 1472-6947-7-3-S13.gz › rap/doc/phpdoc/__filesource/fsource_dataset__syntaxTriXParser.php.html]

xml version="1.0" encoding="iso-8859-1"?


File Source for TriXParser.php


# Source for file TriXParser.php

Documentation is available at TriXParser.php

```
1. <?php
2. // ----------------------------------------------------------------------------------
3. // Class: TriXParser
4. // ----------------------------------------------------------------------------------


8. /**
9. * Temporary implementation of a TriX-Parser (Usable only with PHP > V5)
10. * Currently, it doesn't support any namespaces and has problems with typed literals.
11. * So this parser only works with TRIX documents where the default namespace is the TRIX namespace.
12. *
13. * <BR><BR>History:<UL>
14. * <LI>05-07-2005                : First version of this class.</LI>
15. *
16. * @version  V0.9
17. * @author Daniel Westphal (http://www.d-westphal.de)
18. *
19. * @package     dataset
20. * @access    public
21. ***/
22. class TriXParser
23. {
24. /**
25. * Reference to the graphSet
26. *
27. * @var        GraphSet
28. * @access    private
29. */
30. var $graphSet;

32. /**
33. * Constructor
34. * Needs a reference to a graphSet
35. *
36. * @param    GraphSet
37. * @access    public
38. */
39. function TriXParser(&$graphSet)
40. {
41. $this->graphSet=&$graphSet;
42. }

44. /**
45. * Parse an XML string
46. *
47. * @param   string
48. * @access    public
49. */
50. function parseString($string)
51. {
52. $this->_populateGraphSet(simplexml_load_string($string));
53. }

55. /**
56. * Parse from a file
57. *
58. * @param   string
59. * @access    public
60. */
61. function parseFile($pathToFile)
62. {
63. $this->_populateGraphSet(simplexml_load_file($pathToFile));
64. }

66. /**
67. * Populates the graphSet with namedGraphs and triples.
68. *
69. * @param   object simpleXMLModel  $xmlModel
70. * @access    private
71. */
72. function _populateGraphSet(&$xmlModel)
73. {
74. $defaultGraphOccurred=false;

76. foreach ($xmlModel->graph as $graph)
77. {
78. if (isset($graph->uri))
79. {
80. $graphName=(string)$graph->uri;
81. $namedGraph=& $this->graphSet->getNamedGraph($graphName);
82. if ($namedGraph ==null)
83. $namedGraph=& $this->graphSet->createGraph($graphName);
84. } else
85. {
86. if ($defaultGraphOccurred)
87. trigger_error('Only one unnamed Graph per file allowed', E_USER_ERROR);

89. $namedGraph=& $this->graphSet->getDefaultGraph();
90. $defaultGraphOccurred=true;
91. }

93. foreach ($graph->triple as $triple)
94. {
95. $tripleCount=0;
96. $tripleArray=array();
97. foreach ($triple->children() as $tag => $value)
98. {
99. $tripleArray[$tripleCount++]=$this->_element2Resource((string)$tag,$value);
100. };
101. $namedGraph->add(new Statement($tripleArray[0],$tripleArray[1],$tripleArray[2]));
102. };
103. };
104. }

106. /**
107. * return a mathing resource tyoe
108. *
109. * @param   string
110. * @param   object simpleXMLNode $value
111. * @access    private
112. */
113. function _element2Resource($tag,$value)
114. {
115. switch ($tag)
116. {
117. case 'uri':
118. return new Resource((string)$value);
119. break;

121. case 'id':
122. return new BlankNode((string)$value);
123. break;

125. case 'typedLiteral':
126. $literal=new Literal((string)$value);
127. $literal->setDatatype((string)$value['datatype']);
128. return $literal;
129. break;

131. case 'plainLiteral':
132. $literal=new Literal((string)$value);
133. if(isset($value['xml:lang']))
134. $literal->setLanguage((string)$value['xml:lang']);
135. return $literal;
136. break;
137. }
138. }
139. }
140. ?>
```

Documentation generated on Thu, 7 Jul 2005 13:43:59 +0200 by phpDocumentor 1.3.0RC3
